# Oat evolution revealed in the maternal lineages of 25 *Avena* species

**DOI:** 10.1038/s41598-018-22478-4

**Published:** 2018-03-09

**Authors:** Yong-Bi Fu

**Affiliations:** Plant Gene Resources of Canada, Saskatoon Research and Development Centre, Agriculture and Agri-Food Canada, 107 Science Place, Saskatoon, SK S7N 0X2 Canada

## Abstract

Cultivated hexaploid oat has three different sets of nuclear genomes (A, C, D), but its evolutionary history remains elusive. A multiplexed shotgun sequencing procedure was explored to acquire maternal phylogenetic signals from chloroplast and mitochondria genomes of 25 *Avena* species. Phylogenetic analyses of the acquired organelle SNP data revealed a new maternal pathway towards hexaploids of oat genome evolution involving three diploid species (*A. ventricosa*, *A. canariensis* and *A. longiglumis*) and two tetraploid species (*A. insularis* and *A. agadiriana*). Cultivated hexaploid *A. sativa* acquired its maternal genome from an AC genome tetraploid close to *A. insularis*. Both AC genome *A. insularis* and AB genome *A. agadiriana* obtained a maternal genome from an ancient A, not C, genome diploid close to *A. longiglumis*. Divergence dating showed the major divergences of C genome species 19.9–21.2 million years ago (Mya), of the oldest A genome *A. canariensis* 13–15 Mya, and of the clade with hexaploids 8.5–9.5 Mya. These findings not only advance our knowledge on oat genome evolution, but also have implications for oat germplasm conservation and utilization in breeding.

## Introduction

Oat (*Avena* L., Poaceae family) is one of the most cultivated cereals worldwide and a valuable resource in several countries both for human consumption and animal feed^1^. It also is an interesting model for evolutionary research on the evolution of polyploids. The genus *Avena* contains up to 30 recognized species in a series of ploidy levels that includes diploids, tetraploids, and hexaploids^2,3^. Genome tetraploidization and hexaploidization also occurred in oat species. The cultivated hexaploid oat has 42 chromosomes representing three different sets of nuclear genomes (A, C, D)^4^. However, little is known about the evolutionary history of oat species and oat genome evolution^5^. Many questions about the oat genome evolutions remain unanswered^6–8^. Which diploid species are the genome donors for oat polyploidization? Which tetraploid species are the genome donors for hexaploid oats? When and how frequent did the tetraploidization and hexaploidization events occur? What is the evolutionary pathway leading to hexaploid oat? These questions are not only interesting for evolutionary research, but also are relevant to germplasm conservation and utilization in plant breeding^9,10^.

The last 50 years have seen considerable efforts made to assess *Avena* genome relationships, and the revealed relationships have been extensively documented and discussed^4,5,7,8,11,12^. Hexaploid oat has been proposed to be originated from the formation of an AC genome tetraploid from A and C genome diploids, followed by hybridization to a D genome diploid, with chromosome doubling at each stage to stabilize chromosome pairing^5,8,13,14^. However, this proposal does not specify the genome donors in oat genome origins with solid scientific evidence. More importantly, B and D genomes have not been identified or found in extant diploid oat species^7^. Three AC genome tetraploids closely related to hexaploids are challenged for their genome designations by new evidence from molecular analysis^15,16^, meiotic chromosome pairing^17^, and FISH analysis^18^, and they may contain the D genome found in hexaploids^4^. Consequently, AC genome should be designed as CD or A’C genome^19^. Poorly known is about the process and mode of A, C, or D genome evolution within tetraploids and hexaploids after polyploidization^8^. These issues have become the major stimulus for evolutionary queries towards oat evolution^4,8,19^.

Many experimental approaches have been applied with variable levels of success to search for informative phylogenetic signals for understanding oat evolution^10,12,18–22^. They include ecotype analysis, interspecific crossing, cytogenetic analysis, gene-specific inference, and molecular marker application, and each approach has played a role in the inference of oat evolution^7^. These applications, particularly with molecular markers, have helped to elucidate the species relationships of the genus *Avena*^5^. The most informative phylogenetic analyses are those based on the alignments and comparison of conserved nuclear or chloroplast genes, allowing for concise estimates of the number and order of genetic mutations within and between species. For example, Liu *et al*. revealed an interesting evolutionary dynamics of ancient and recent polyploidization events in *Avena* based on three nuclear genes and three plastid genes^19^. However, these studies also carried some limitations. First, single genes may not represent the average rate of divergence across entire genomes, and may not show adequate diversity to resolve differences within a species or between closely-related species^4^. Second, gene-based phylogenetic inference can be less informative with variable ancestral population size^23^ and suffered from incomplete lineage sorting^24^. Third, many studies also have had sampling issues with species^25^ and genomic^4^ coverages.

The earliest search for organelle (chloroplast, cp; mitochondria, mt) phylogenetic signals in oat species was made by Steer *et al*.^26^, followed by Murai and Tsunewski^27^ and Rines *et al*.^28^. Recent years have seen some researches in the acquisition of phylogenetic signals from cp genomes^19,22,29^. These researches have helped to provide insights into maternal origins of oat genomes, confirming the general consensus in oat phylogeny^4,19^. However, no studies have been reported taking advantage of next generation sequencing technology to explore genome-wide variability of oat organelle genomes for phylogenetic inferences^30^. Here we explore a multiplexed shotgun sequencing procedure to acquire maternal phylogenetic signals from cp and mt genomes of 25 *Avena* species. The specific objectives of this study were to elucidate maternal phylogenetic relationships of 25 *Avena* species, assess maternal pathway of A, C and D genomes towards hexaploid oat, and infer the history of major divergences in oat species.

## Materials and Methods

### Plant materials

We selected 25 *Avena* accessions of known species identity from the Plant Gene Resources of Canada (PGRC) oat collection based on our previous oat research^4,25^. The selected accessions originated from various regions around the world and represent 25 species of the six botanical sections of the *Avena* genus: *Ventricosa*, *Agraria*, *Tenuicarpa*, *Pachycarpa*, *Ethiopica*, and *Avena*, and five distinct nuclear genomes organized in diploid (A or C), tetraploid (AB or AC) and hexaploid species (ACD). To avoid confusion on genome designation, we will continue to use AC, rather CD, genome for AC tetraploids^4^ in this study. Table 1 shows the detailed information on the selected accessions, including botanical section, ploidy, and genome designation. About 300 seeds from each accession were planted in August 2013 in a 15 cm pot, grown for 8 to 10 days, and then incubated in the dark for 48 to 72 hours in the greenhouse at Saskatoon Research and Development Centre. Up to 15 g of all 300 seedling leaves were collected and washed in cold water. Leaves were cut into 1 cm pieces with scissors, snap frozen with liquid nitrogen in a −20 °C mortar, and ground to a fine powder. Ground samples, while still frozen, were transferred to 50 ml conical bottom centrifuge tubes, cooled on dry ice, and then stored at −80 °C for up to one week.

**Table 1:** List of 25 studied *Avena* species from six botanical sections and their ploidy, genome, sample, origin of country, sequencing and SNP information.

Section/Species	Ploidy	GD^a^	S-CN^a^	Origin^a^	Seq^a^	Total^a^	cp^a^	mt^a^	uScp/mt^a^	Label
Ventricosa										
*A. ventricosa*	2×	Cv	21992	Cyprus	R3S5	3399167	1684406	78839	289/311	ventricosa_Cv
*A. clauda*	2×	Cp	19205	Iran	R4S3	3037449	1924742	84662	154/129	clauda_Cp
*A. eriantha*	2×	Cp	19256	Iran	R2S4	3932778	2378444	123230	254/372	eriantha_Cp
Agraria										
*A. hispanica*	2×	As	25788	Portugal	R4S6	3138274	1893975	102706	117/149	hispanica_As
*A. brevis*	2×	As	3145	Portugal	R4S7	3145903	1869416	117171	105/138	brevis_As
*A. nuda*	2×	As	79350	UK	R4S5	3342392	2223962	74936	89/129	nuda_As
*A. strigosa*	2×	As	22002	Uruguay	R4S4	2618401	1715256	73544	119/130	strigosa_As
Tenuicarpa										
*A. canariensis*	2×	Ac	25449	C-Islands	R3S6	4285394	2454563	128936	287/247	canariensis_Ac
*A. damascena*	2×	Ad	19458	Syria	R1S6	2906067	1881077	79666	167/188	damascena_Ad
*A. atlantica*	2×	As	25859	Morocco	R1S4	4092127	2055044	86104	121/161	atlantica_As
*A. wiestii*	2×	As	24315	Israel	R2S5	4372976	2696750	143105	150/218	wiestii_As
*A. lusitanica*	2×	As	25936	Morocco	R2S6	4425054	2557961	151205	156/182	lusitanica_As
*A. longiglumis*	2×	Al	21407	Algeria	R1S5	4288420	2836725	90183	212/200	longiglumis_Al
*A. agadiriana*	4×	AB	25868	Morocco	R2S3	4432963	2826976	142278	148/217	agadiriana_AB
*A. barbata*	4×	AB	24462	Turkey	R2S2	4362663	2757979	141784	117/163	barbata_AB
Pachycarpa										
*A. insularis*	4×	AC	19178	Italy	R3S4	4001844	2397235	108973	212/307	insularis_AC
*A. maroccana*	4×	AC	23057	Morocco	R4S2	4160684	2688390	125733	117/156	maroccana_AC
*A. murphyi*	4×	AC	21989	Spain	R3S3	5199242	3202765	129771	202/273	murphyi_AC
Ethiopica										
*A. vaviloviana*	4×	AB	22413	Ethiopia	R1S2	3870454	2400339	122730	161/199	vaviloviana_AB
*A. abyssinica*	4×	AB	22064	Ethiopia	R1S3	2822158	1744923	80606	195/257	abyssinica_AB
Avena										
*A. fatua*	6×	ACD	21948	Ethiopia	R1S1	4120412	2700471	101994	111/161	fatua_ACD
*A. hybrida*	6×	ACD	24926	Iran	R2S1	4330653	2815803	118441	147/179	hybrida_ACD
*A. occidentalis*	6×	ACD	25946	Morocco	R4S1	2947248	2033154	60953	115/134	occidentalis_ACD
*A. sterilis*	6×	ACD	20625	Israel	R3S1	2467386	1473445	64418	229/262	sterilis_ACD
*A. sativa*	6×	ACD	24549	Turkey	R3S2	5329202	3377953	119617	161/228	sativa_ACD
*Mean*						3801172	2343670	106063	165/204	

^a^GD stands for genome designation. S-CN is sample label with Canadian National accession number at the Plant Gene Resources of Canada (PGRC), Saskatoon, Canada. Origin for a sample is the country of origin; C-Islands is Canary Islands. Seq is for sequencing done with four Illumina MiSeq runs, each having six or seven multiplexed barcoded samples; R short for run and S for sample label. Total is the total number of sequence reads obtained for a sample. cp represents the count of chloroplast genome sequence reads. mt is for the count of mitochondrial genome sequence reads. uScp/mt stands for unique cp and mt SNP counts for each sample, respectively.

### Plastid DNA isolation

The procedure for isolating oat plastid DNA was developed mainly following two published chloroplast DNA extraction methods^31,32^. It was optimized for specific oat species and extensively tested before its application for this study. All steps of the plastid DNA isolation were carried out on ice or at 4 °C with buffers pre-chilled to 4 °C. Frozen ground leaf tissue was transferred to a beaker containing 190 ml of Buffer A^31^ and a magnetic stir bar. The sample was stirred at a moderate speed for 15 min to evenly distribute the tissue throughout the buffer. The homogenate was filtered through two layers of Miracloth (Calbiochem, EMD Chemicals, San Diego, USA) into a second beaker to remove solid tissue debris from the liquid filtrate and then the Miracloth was squeezed by hand to maximize liquid recovery. To remove fine debris, the filtrate was filtered a second time through four layers of Miracloth, without squeezing, into a 250 ml centrifuge bottle and centrifuged using a fixed angle rotor at 1500 × g for 20 min. The supernatant was gently poured off, leaving the resulting loose green crude chloroplast enriched pellet.

The following steps to clean the crude chloroplast pellet were carried out in 50 ml conical bottom centrifuge tubes using a swinging bucket rotor. All resuspensions were done gently using a No. 4 round paint brush. The pellet was resuspended in 45 ml of Buffer B^31^ and centrifuged at 3500 × g for 20 min. The supernatant was poured off and the pellet was resuspended in 40 ml of Buffer B and centrifuged at 3750 × g for 20 min. The supernatant was poured off and the pellet was resuspended in 5 ml of Wash Buffer^32^, followed by loading on top of a 30 ml 30% sucrose cushion^32^ and centrifuging at 2500 × g for 20 min. The supernatant was poured off and the cleaned chloroplast enriched pellet was rinsed, without resuspension, by adding 40 ml of Wash Buffer and centrifuging at 2500 × g for 5 min. The supernatant was poured off and the resulting pellet was resuspended in 1.5 ml of Wash Buffer, transferred to a 2 ml centrifuge tube, and centrifuged at 380 × g for 5 min in a bench top microcentrifuge to pellet chloroplasts. The supernatant was pipetted off and the cleaned chloroplast enriched pellet was snap frozen in liquid nitrogen.

The pellet was allowed to thaw at room temperature and DNA was extracted using the Qiagen DNEasy Plant Mini kit standard method on a Qiacube robot (Qiagen, Mississauga, Canada) and eluted in 1/3× Qiagen AE buffer (3.33 mM Tris-Cl, 0.17 mM EDTA, pH 9.0). Extracted DNAs were quantified using the Quant-iT PicoGreen dsDNA Assay Kit (Life Technologies, Burlington, Canada). Final DNAs yielded from 0.2 to 4.3 ng/µl and were diluted to 0.2 ng/µl with 10 mM Tris-HCl, pH 8.

### Multiplexed shotgun sequencing

Illumina MiSeq shotgun sequencing libraries were prepared using 1 ng of plastid DNA with the Nextera XT DNA Library Preparation Kit (Illumina, San Diego, USA) with Nextera XT Index Kit dual indexed adapters. Sample concentration normalization was completed using the Quant-iT PicoGreen dsDNA Assay Kit, rather than the Nextera XT bead-based normalization step. Quantified samples were diluted to 4 nM with 10 mM Tris-HCl with 0.1% Tween 20, pH 8.5. Six to seven libraries, each with a unique pair of indexes, were combined into a pooled library. As nuclear genome contamination existed and varied in ploidy from diploid to hexaploid, each pool was designed with proper sample combination for a maximum of 24 haploid genomes in total to keep the nuclear genome contamination consistent to minimize the sequencing bias across four MiSeq runs. Five µl of each of 6–7 samples with a unique pair of indexes were combined in a 1.5 ml microcentrifuge tube and mixed on a shaker for 1 min at 1800 rpm. The pooled libraries were denatured according to the MiSeq Reagent Kit (v3, 600 cycles) protocol and diluted to a final concentration of 6–9 pM with a spike-in of 1% Illumina PhiX. Four MiSeq runs with paired-ends of 250 bp in length were made in March-April 2014 and generated 25 forward and 25 reverse FASTQ files. These raw sequences were deposited into NCBI’s SRA database under BioProject ID of PRJNA401438.

### Bioinformatics analysis for SNP data

The original research was conceived in 2013 with sufficient cp genome coverage to acquire oat phylogenetic signals from cp sequences only. The development of 3GenomeSNP pipeline in 2015 for generating genome-wide SNP data from organelle and nuclear genomes from MiSeq shotgun sequences of a non-model organism^30^ opened an opportunity to explore phylogenetic signals from other genomes. An exploratory application of the 3GenomeSNP pipeline revealed an unexpected outcome that substantial mt and nuclear genome sequences were also present in these oat FASTQ files (see Table 1 for mt sequence reads). Thus, the FASTQ sequence data were analyzed to generate both cp and mt SNP data per sample for phylogenetic analysis.

The complete bioinformatics analysis was performed with four major steps. First, an extraction was made of sequence reads from each FASTQ file into two separate FASTQ files with unique cp or mt sequences. This was done with a modification to the 3GenomeSNP pipeline for generating genome-wide SNP data from organelle genomes alone. Specifically, Bowtie2 software with the–al-conc flag^33^ was applied to map sequence reads to the reference organelle genomes and to extract those mapped sequence reads into a separate FASTQ file. There are no oat organelle genomes sequenced and reported so far^34^. In this analysis, wheat cp genome sequences (GenBank accession: AB042240)^35^ and wheat mt genome sequences (GenBank accession: AP008982)^36^ were used as reference genomes, as wheat is the most closely related grass species with both cp and mt genome sequences published. To remove the possible repeat sequence reads between the cp and mt reads in each sample, the cp and mt reads were aligned against each other using Bowtie2 with the–un-conc flag and custom shell and Perl scripts. Sequence reads unique to cp or mt were generated and saved into separate FASTQ files for each sample. Second, a specific shell script was developed to do parallel computing for generating sorted.bam file from each FASTQ file and to perform SNP calling from all BAM files across 25 samples. This was done first with mapping the separated organelle sequence reads in each sample against the corresponding wheat organelle genome sequences using Bowtie2 to generate a SAM file. Based on the SAM file, SAMtools^37^ was used to create, sort and index BAM files for SNP calling. SNP calls were conducted using the ANGSD pipeline^38^ with the parameter settings given in Electronic Supplementary Material (ESM), and they were based on allele frequencies that were estimated with genotype likelihoods from the sorted.bam files. This procedure was done separately for cp or mt FASTQ files. Third, another specific shell script was developed to do parallel counting of total sequences and those sequences unique to cp and mt for all original and separated FASTQ files. Fourth, the SNP loci with missing values across 25 samples were removed from further analysis to avoid the possible complication to phylogenetic inferences, particularly of divergence dating. The frequency distributions of the detected cp and mt SNPs across the wheat organelle genomes were calculated using a custom R script^39^. The minor allele frequency at each cp or mt SNP locus across 25 species and non-informative SNPs per sample were calculated in Microsoft Excel^®^. To facilitate the phylogenetic analysis below, we also converted each FASTA SNP data set to the related NEXUS or PHYLIP formats using either ClustalX 2.0^40^ or PGDSpider v2.1.0.0^41^.

### Phylogenetic analysis

Three types of phylogenetic analysis were performed in a Linux server based on cp, mt and combined (or cpmt) SNP data sets of 25 *Avena* samples using BEAST v2.0.3^42^, MrBay 3.2.6^43^, RAxML v8.2.7^44^ and/or PAUP* 4.0^45^ software. First, the phylogenetic relationships of 25 *Avena* species were inferred through Bayesian, maximum likelihood (ML), and maximum parsimony (MP) approaches. Specifically for each data set, a Bayesian maximum clade credibility (MCC) tree with an outgroup of wheat and posteriori probability support was generated using BEAST with the parameter and option settings described in ESM. Similarly, a Bayesian MB tree with an outgroup of wheat and posteriori probability support was acquired using MrBay with the parameter and option settings described in ESM. A ML tree with bootstrap support was produced using RAxML with the K80 substitution model (-m GTRCAT -V–K80) and with the executed parameters and options shown in ESM. A MP tree with bootstrap support was obtained using PAUP* with the parameters and options listed in ESM. Note that the options and parameters used above to generate MCC, MB, ML and MP trees were obtained from large training trials for each method, and that multiple phylogenetic analyses of the same data set would allow for comparative assessments of data consistency in tree topology.

Second, specific phylogenetic analyses with evolutionary questions were made using specific sets of *Avena* species to infer oat cytoplasm origins based on oat genome relationships. Based on the results from the first set of phylogenetic analyses above, we selected three smaller sets of *Avena* samples to address the following questions: (1) Is A or C genome closer to ACD genomes *A. sativa* and *A. sterilis*? (2) which A genome is closer to AC genome *A. insularis* or AB genome *A. agadiriana*? and (3) Is AC or AB genome closer to ACD genomes *A. sativa* and *A. sterilis*? For each question with specific data set, MCC, MB, ML, and MP radiation trees were generated, without considering the outgroup, using the same approaches or parameters given in ESM for BEAST, MrBay, RAxML and PAUP* software. Preliminary analyses revealed that smaller sets of oat samples had better resolutions than the entire oat samples for the comparative assessments of genome relatedness and topological uncertainty. To visualize the topological uncertainties in inferences of oat genome relationship, Bayesian density trees were also generated using DensiTree 2.2.6 software^46^ from the MCC trees that were generated using BEAST software with the same settings as described in ESM.

Third, a Bayesian inference of divergence times among 25 *Avena* species was made using BEAST software with the parameter settings listed in ESM. The inference was done separately for each data set. A modification was made for running for cpmt data set with a tree prior of calibrated Yule model. The K80 model of nucleotide substitution without rate heterogeneity was the best fit for the concatenated SNP data from genotyping-by-sequencing or restriction site associated DNA tag techniques when determined using jModelTest^47,48^ and further confirmed from the extensive training trials in this study. The convergence of parameters among runs was evaluated visually using Tracer v.1.6^49^. The output tree files were loaded into TreeAnnotator in the BEAST package with the default options: 10% burnin and 0.50 posteriori probability limit and median node heights to combine and construct a MCC tree. The Figtree_v1.4 software (http://tree.bio.ed.ac.uk/software/figtree/) was used to display the MCC tree with the posterior probability as branch support and with node labels with time scale to root age of 25 million years for the estimated divergence between wheat and oat^50^.

## Results

### Sequence reads and SNP discovery

Four MiSeq runs generated a total of 95,029,311 sequence reads of 250 bp in length, among which 58,591,754 sequences were unique to cp and 2,651,585 sequences were unique to mt (Table 1). Specifically, each sample for a given species had an average of 2,343,670 unique cp sequences and 106,063 mt sequences, giving a roughly 4300× cp genome coverage based on wheat cp genome length and 59 ×mt genome coverage based on wheat mt genome length. SNP calls from these sequences for 25 samples generated a total of 6,694 cp SNPs and 17,874 mt SNPs. There were 6329, 6476, and 6536 cp SNPs with 0, 10%, and 30% level of missing values or less, respectively. Similarly, there were 6343, 8256, and 11,118 mt SNPs with 0, 10%, and 30% level of missing values or less, respectively. Evaluating the genome-wide distribution of the SNPs without missing values showed both cp and mt SNPs were widely distributed across the wheat cp and mt genomes, respectively (Fig. S1 in ESM). Analyzing the minor allele frequencies of the SNPs without missing values revealed 4135 (65.3%) cp SNPs, and 5090 (80.2%) mt SNPs with an allele present in only one out of 25 samples (or allele frequency of 0.04) (Fig. S2). The counts of such non-informative cp and mt SNPs for each sample are given in Table 1, and on average, each sample had 165 non-informative cp SNPs and 204 non-informative mt SNPs. There were 2194 cp and 1243 mt parsimony-informative SNPs with alleles having higher frequency up to 0.46 (Fig. S2), allowing for informative phylogenetic inferences of 25 oat species.

### Phylogeny

**Figure 1:**
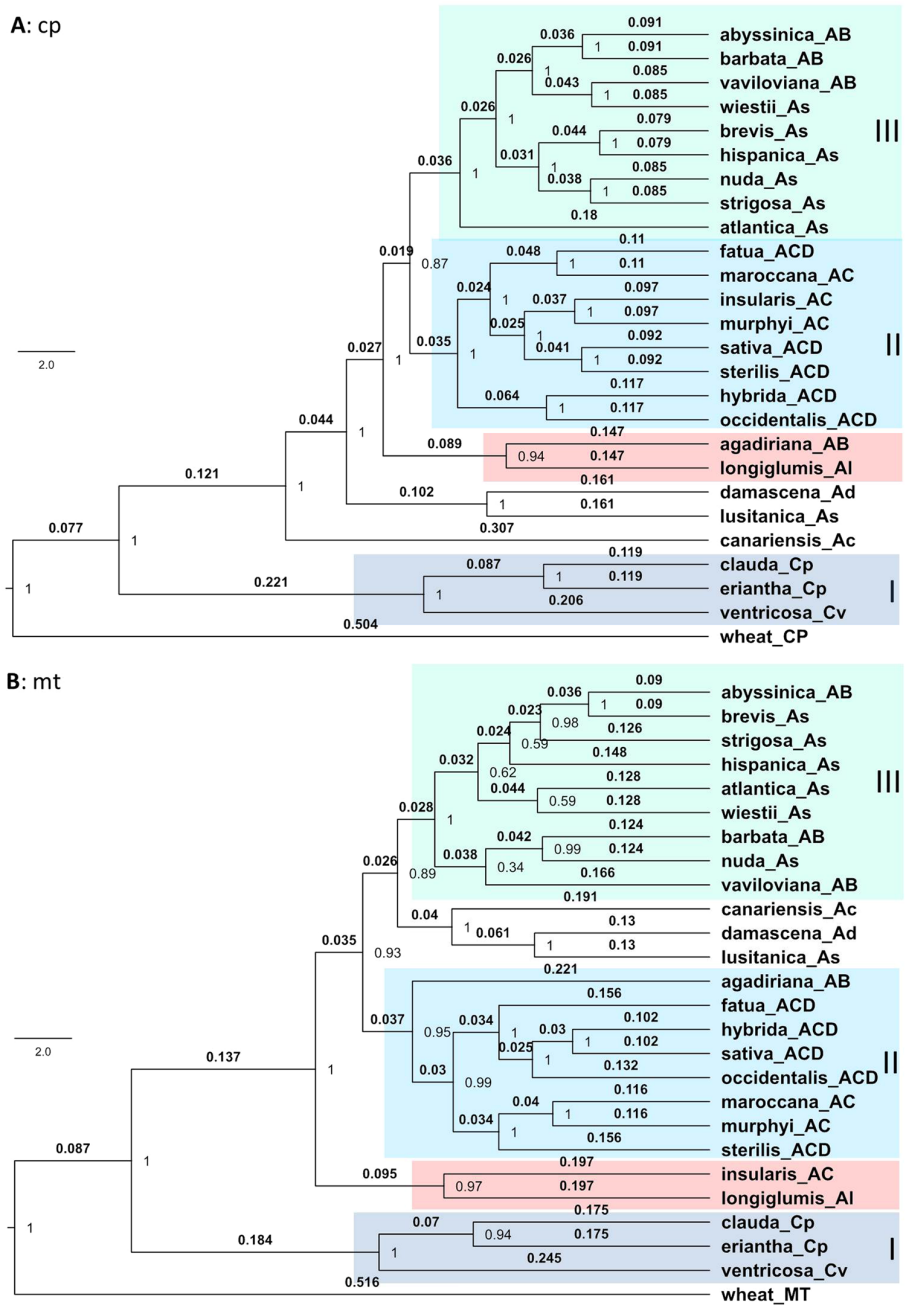
Phylogenetic trees of 25 oat species with branch length and support and with wheat as an outgroup. They were inferred using BEAST software based on 6329 chloroplast (cp) (**A**) and 6343 mitochondrial (mt) (**B**) SNP data sets. The branch lengths are shown above the branches and branch supports at nodes are posterior probability. Three major clades (I, II, III) are highlighted and labeled on each tree. The branch for *A. agadiriana* and *A. longiglumis* closest to Clades II and III is also highlighted.

Maternal phylogenetic trees of 25 *Avena* species with branch length, branch support and wheat as outgroup were generated through Bayesian, maximum likelihood and maximum parsimony approaches on three data sets. It was found that the Bayesian approaches generated phylogenetic trees with much higher branch supports than the ML and MP approaches (Figs 1 and S3). Also, the Bayesian MCC trees carried important parameter estimates such as clade ages and branch lengths. Thus, the MCC trees will be focused for the interpretation of the maternal phylogeny below, while the phylogenetic trees inferred from other methods will be compared for the interpretation of poorly resolved branches and for the assessment of lineage uncertainties.

Figure 1 shows two Bayesian MCC trees of 25 *Avena* species with branch length and support obtained using BEAST software from cp and mt SNP data sets. Examining these two MCC trees revealed several patterns. First, three Ac genome species of section *Ventricosa* were substantially diverged from the other species, consistently forming their own sectional clade I across two data sets. Clearly, *A. clauda* and *A. eriantha* were more closely related each other than *A. ventricosa*. Second, the eight species of section *Tenuicarpa*, including A and AB genomes, did not have their own sectional clade, but formed a large clade with As, AC and ACD genome species. Within this large clade, two major clades were also observed. The four As genome species of section *Agraria* had their own small sectional clade based on cp data, but they also were a part of the distinct clade III with some A genome species of Section *Tenuicarpa* and two AB genome species of section *Ethiopia*. Similarly, three AC genome species of section *Pachycarpa* were closely related to five ACD genome species of section *Avena*, together forming a distinct clade II based on cp data. Third, two species of section *Tenuicarpa*, *A. agadiriana* and *A. longiglumis*, are interesting, as both were more closely related to the AC-ACD genome species than the other A or AB genome species. More interestingly, Al genome *A. longiglumis* was closely related to AB genome *A. agadiriana* based on cp data and to AC genome *A. insularis* based on mt data. Fourth, *A. fatua*, *A. hybrida* and *A. occidentalis* displayed distinct maternal origins separate from *A. sativa* within the clade II based on cp data. *A. agadiriana* showed clear separation from the other AB genome species. *A. lusitanica* was distantly related to the other As genome diploids. Fifth, the branch supports for these phylogenetic trees are generally high, ranging from 87 to 100 based on cp data and from 34 to 100 based on mt data. The cp-based tree had the better branch supports than the mt-based tree in which the clade III displayed a few branches with low supports.

Topological comparisons among the phylogenetic trees obtained from four different phylogenetic methods for different data sets (e.g. see Figs 1 and S3) revealed nearly the same topologies as observed in, and described for, the MCC trees above. Across all the phylogenetic trees, three major clades largely remain, and the distinct feature of *A. agadiriana* and *A. longiglumis* of section *Tenuicarpa* is consistently displayed. Similarly, cp-based and mt-based trees, even with marked differences in branch supports, still are largely compatible in tree topology.

### Specific genome pathway

**Figure 2:**
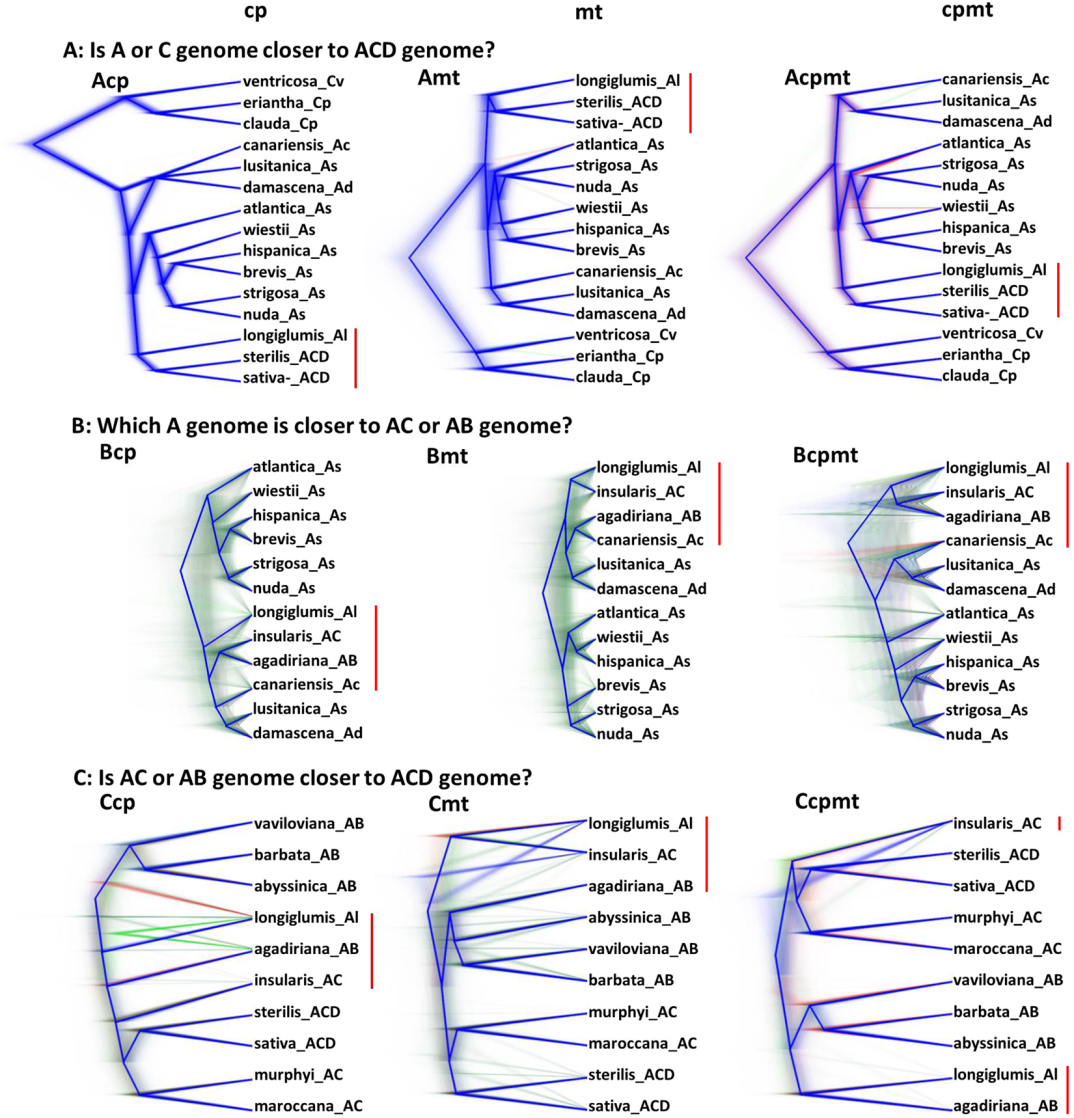
Oat genome relationships as revealed with the Bayesian density trees for three specific groups of oat species. They were inferred using BEAST software based on 6329 chloroplast (cp), 6343 mitochondrial (mt) and 12,672 combined (cpmt) SNP data sets. The major concerned species are highlighted in red bar among three data sets for each set of oat samples for ease of comparisons.

Three questions for cultivated ACD genome pathway were addressed through phylogenetic analyses of specific sets of oat samples using BEAST, MrBay, RAxML and PAUP* software. The Bayesian DensiTrees from the BEAST analysis are displayed in Fig. 2, and the other phylogenetic trees are presented in Figs S4–S6. These specific DensiTrees are more informative to visualize lineage uncertainties than a DensiTree with all 25 oat species. The first question is whether the origin of the cytoplasm of the ACD genome species is from the A or C genome species, and all three C genome and 10 A genome species were selected along with two closely related ACD genome species *A. sativa* and *A. sterilis*. The Bayesian DensiTrees (Fig. 2A) from three data sets showed (1) that A genome species were more related to *A. sativa* than C genome species and (2) that *A. longiglumis* was the most closely related A genome species to *A. sativa*. Similarly, *A. ventricosa* appeared to be a little closer than the two other C genome species to *A. sativa*. These results are further confirmed by those from MrBay, RAxML and PAUP* analyses (Fig. S4). The second question is on the relatedness of the cytoplasm of A genome to AC or AB genome, and all the A genome species were selected along with the AC genome *A. insularis* and the AB genome *A. agadiriana*. The results showed that Ac genome *A. canariensis* or Al genome *A. longiglumis* was closer to tetraploids of *A. insularis* and *A. agadiriana* than the other A genome diploids based on cp data (Fig. 2Bcp). Based on mt data, Ac genome *A. canariensis* was closer to *A. agadiriana*, while Al genome *A. longiglumis* was closer to *A. insularis* (Fig. 2Bmt). Based on combined cpmt data, Ac genome *A. canariensis* was not closed to *A. insularis* and *A. agadiriana* (Fig. 2Bcpmt). These results were compatible with those obtained from the other three methods (Fig. S5). The third question is on the relatedness of the cytoplasm of AC or AB genome species to the ACD genome species, and all three AC genome and four AB genome species were selected along with the related Al genome and two ACD genome species. The DensiTrees (Fig. 2C) from three data sets provided support for the closer relations of AC genome species than AB genome species to *A. sativa*. AB genome *A*. *agadiriana* was more related to AC genome species than the other AB genome species, based on cp data (Fig. 2Ccp), but such close relatedness was not supported from mt or cpmt data sets (Fig. 2Cmt and Fig. 2Ccpmt). Interestingly, the cpmt data showed the close relatedness between AB genome *A*. *agadiriana* and Al genome *A. longiglumis*. These results were consistent with those obtained from the other three methods (Fig. S6).

### Divergence dating

**Figure 3:**
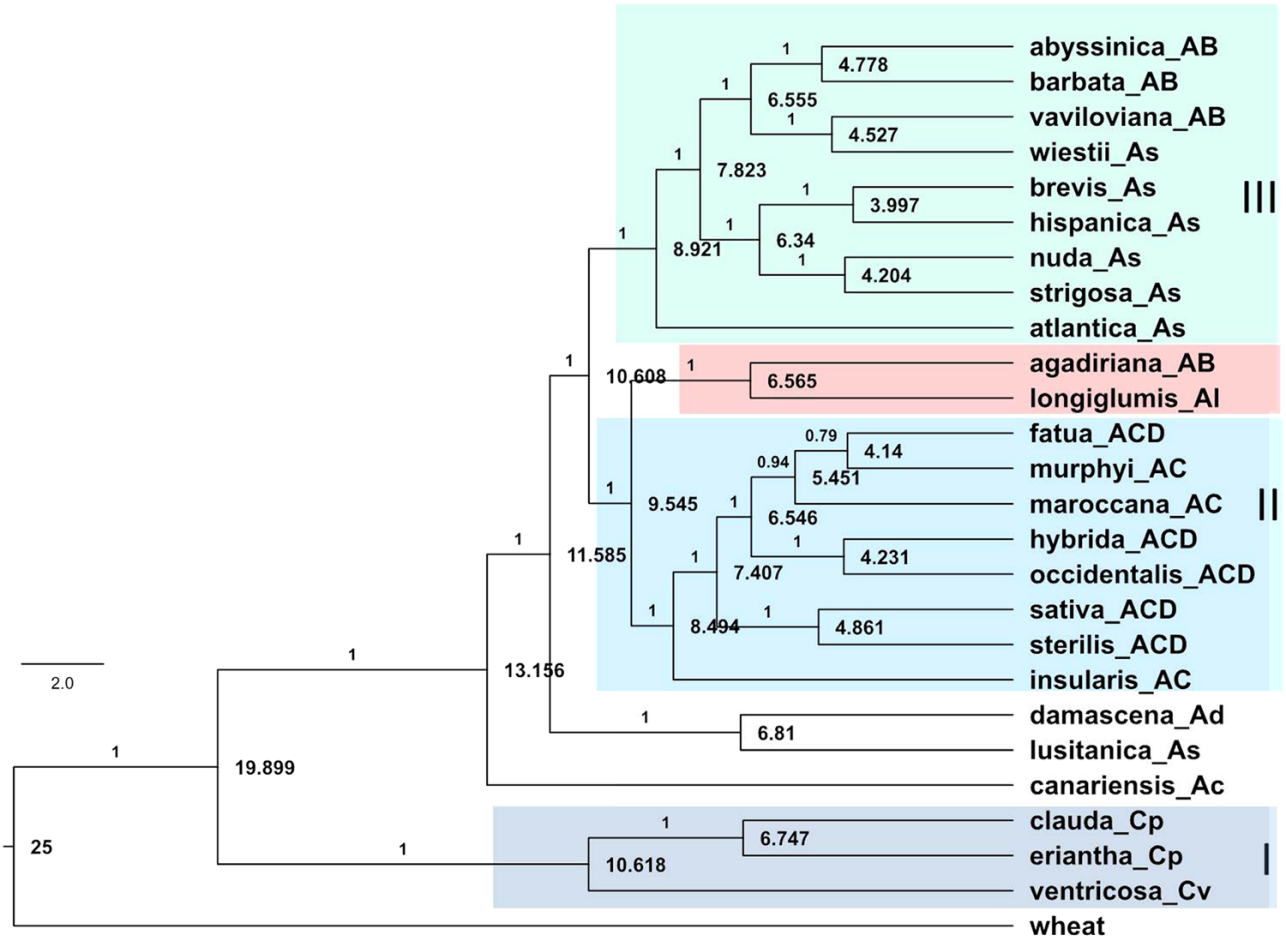
Phylogenetic trees of 25 oat species with branch support, node age and an outgroup of wheat. These MCC trees were obtained using BEAST software based on 12,672 combined cp and mt SNP data set. The values above the branch are posterior probability and the node ages are calibrated with the wheat-oat divergence of 25 Mya. Three major clades (I, II, III) are highlighted and labelled. The branch for *A. agadiriana* and *A. longiglumis* closest to Clade II is also highlighted.

Divergence dating was made specifically using BEAST software based on three data sets, after extensive Bayesian analysis made to find the best-fit models for site model, clock model and priors. No marked differences in divergence time were found between site models of HKY and GTR and between the priors of Yule model and calibrated Yule model. However, the clock model showed the large impacts on the estimation of divergence time, and the model of relaxed clock exponential appeared to be best fit. Based on the best-fit model, running BEAST analysis generated three Bayesian MCC trees for three data sets. Essentially, the same topologies of 25 *Avena* species generated for each data set were obtained as those from the three other methods as described above. Figure 3 shows the MCC tree with branch support, node age and outgroup of wheat, based on combined cpmt data. Calibrated with the wheat-oat divergence of 25 million years ago (Mya), C genome oat species diverged around 20 Mya from the remaining oat species. *A. ventricosa* separated from *A. clauda* and *A. eriantha* around 10–11 Mya. The oldest A genome species was *A. canariensis*, separated from the other A genome species around 13–15 Mya. Another possible A genome species contributing to the clade II with AC and ACD genomes was *A. longiglumis* with an estimate of age 12 Mya. Also, the separation between clade III (with As and AB genomes) and clade II (AC and ACD genomes) was estimated to occur 11 Mya. Within the clade III, the divergence times for the nine species ranged from 4 to 8.9 Mya, and *A. atlantica* was the oldest in the clade (Fig. 3). Within the clade II, the eight species diverged from 4.1 to 9.5 Mya. *A. insularis* was the oldest AC genome tetraploid in the clade with an estimated age of 8.5 Mya. Interestingly, the separation of *A. sativa* from *A. sterilis* was estimated to be 4.9 Mya. Divergence dating from the MCC trees based on separate cp or mt data (results not shown) was largely compatible with those described above, and the revealed variations in dating allowed for a rough estimation of the dating range for some lineages. For example, *A. ventricosa* separated from the other two C genome diploids with an estimate of 10.229 Mya from cp data, 11.894 Mya from mt data, and 10.618 Mya from combined data.

## Discussion

Our study presented here represents the first comprehensive characterization of organelle genomic variability in oat species. Based on these maternal genomic signals, the phylogenetic analysis of these 25 *Avena* species revealed several interesting patterns of oat evolution. First, three distinct clades of oat species were found: clade I for three C genome species of section *Ventricosa*; clade II for three AC genome species of section *Pachycarpa* and five ACD genome species of section *Avena*; and clade III for four As genome species of section *Agraria*, some A or AB genome species of section *Tenuicarpa*, and two AB genome species of section *Ethiopica*. Second, the eight species of section *Tenuicarpa*, including A and AB genomes, did not form their own sectional clade, and *A. agadiriana* and *A. longiglumis* were evolutionarily distinct, showing close relations to the AC-ACD genome species. Al genome *A. longiglumis* was closely related to AB genome *A. agadiriana* based on cp data and to AC genome *A. insularis* based on mt data. Third, the cultivated hexaploid oat *A. sativa* appeared to acquire a maternal genome from an A genome diploid probably through an AC genome tetraploid. Fourth, C genome oat species diverged around 20 Mya from the remaining oat species. The oldest A genome *A. canariensis* had separated from the other A genome species for 13–15 Mya. The divergence leading to hexaploid oat occurred 8.5–9.5 Mya. These findings illustrate a new maternal view of oat evolution, advance our knowledge on oat genome evolution, and have practical implications for the conservation of oat wild relatives and the genetic improvement of cultivated oat.

### Oat phylogeny

The maternal phylogeny of 25 oat species obtained in this study appears to deviate little from those obtained from other phylogenetic inferences based on cp DNA markers^19,22,29^, as the major species divergences largely hold. The major differences lie mainly in the branch length and the resolution in some species delimitations. Chloroplast SNP data showed higher resolutions of species divergence than mt SNP data, probably due to the fact that there were more parsimony informative SNPs in cp, than mt, data set. Similarly, our inferences of oat species relationships are compatible with those based on nuclear genomic signals. For example, the genetic associations of 163 individual plants representing 25 oat species based on 413 AFLP markers showed similar major divergences for these species (see Fig. 2 of Fu and Williams^25^). Thus, the maternal phylogeny of 25 oat species is complementary to our current understanding of oat evolution in extant oat species. However, the oat species relationships do not necessarily correlate accurately with the oat genome relationships, as genome can be modified, go distinct, and evolve at a faster rate than speciation, particularly after polyploidization^8^.

### The origin of hexaploid oat

**Figure 4:**
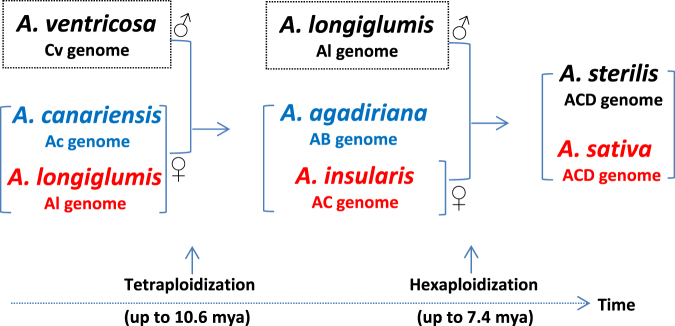
Proposed scenario for the maternal origins of hexaploid oat with the extant *Avena* species based on the organelle phylogenetic signals. Cultivated hexaploid *A. sativa* acquired its maternal genome from an AC genome tetraploid close to *A. insularis*, highlighted in red. Both AC genome *A. insularis* and AB genome *A. agadiriana* obtained a maternal genome from an ancient A genome diploid close to *A. longiglumis* in red. Ac genome *A. canariensis* and AB genome *A. agadiriana*, highlighted in blue, are the oldest living species also likely contributing to the maternal genome of tetraploid and hexaploid, respectively. The paternal genome contributors of diploids to tetraploid and hexaploid are also reasoned based on existing literature in oat evolution in combination with the revealed oat genome relationships. Note that AC or AB genome designations may be renamed as CD genome based on the findings of Yan *et al*.^4^ to minimize the confusion on oat genome evolution. Multiple tetraploidziation and hexaploidization events occurred and are shown with the upper limits of age inferred from the divergence of *A. ventricosa* and the clade II leading to hexaploid oat, respectively (see Fig. 3).

The phylogenetic analyses of specific groups of oat species with different genome designations for specific questions to infer oat genome evolution revealed many interesting maternal patterns of species relatedness in 25 oat species, as illustrated in Figs 2 and S4–S6. Based on these patterns and revealed uncertainties in genome relationship, we reasoned a new evolutionary maternal pathway of extant oat species towards cultivated hexaploid *A. sativa* as a hypothesis to stimulate further evolutionary research. To make the picture of hexaploid oat evolution complete, the paternal genome contributions of diploids to tetraploid and hexaploid were also deducted based on the existing literature in oat evolution (see below) in combination with the revealed oat genome relationships presented here. As illustrated in Fig. 4, cultivated hexaploid *A. sativa* originated first from the tetraploidization event up to 10.6 Mya between a paternal C genome diploid (likely Cv genome *A. ventricosa*) and a maternal A genome diploid close to Al genome *A. longiglumis*, followed by a hexaploidization event up to 7.4 Mya between a paternal diploid species (likely Al genome *A. longiglumis*) and an ancestral, maternal tetraploid close to AC genome *A. insularis*. The two species (*A. canariensis* and *A. agadiriana*), highlighted in blue, represent the oldest living diploid and tetraploid species also likely contributing to maternal genomes of tetraploid and hexaploid, respectively. This pathway for hexaploid oat differs substantially from those proposed pathways based on nuclear phylogenetic signals^5,8,14^, but it should provide a testable model of oat genome evolution with close species relatedness within extant 25 *Avena* species. Precisely, these five species may not all be the immediate genome donors for hexaploid oat, but they represent the important related wild progenitors of various genome types in extant oat species one can find for hexaploid oat, offering guidance for further searching and testing for the true genome donors.

Examining the literature mainly in the nuclear genome origins of hexaploid oat revealed some support for the maternal pathway. First, based on the asymmetrical karyotype with a preponderance of heterobrachial chromosomes species, Rajhathy^51^ proposed that *A. ventricosa* was the C genome donor towards hexaploid oat. Recent studies also provided support for *A. ventricosa* being the ancestral *Avena* genome based on 45 S rDNA external transcribed spacer organization variability^52^ and based on ITS1-5.8 SITS2 sequence variability^53^. Second, *A. canariensis* was the only diploid oat species with denticulate lemma tips, which is a morphological feature in common with hexaploid oat^54^. This species has remarkable morphological variations, distinctive ecotypes, and polymorphic protein and isozyme electrophoretic patterns^55^. Luo *et al*.^56^ found the evidence from fluorescence *in situ* hybridization with probes pITS and A3-19 that Ac genome *A. canariensis* was closely related to the D genome, possibly as the D-genome donor of cultivated oat. Third, Nikoloudakis *et al*.^57^ were able to reveal the close association of *A. longiglumis* to AC and ACD polyploids based on the ITS1-5.8S-ITS2 & IGS nucleotide polymorphism. Similarly, Yan *et al*.^4^ provided direct evidence based on high-density markers profiling showing *A. longiglumis* was the A genome species most closely related to ACD genome species. Fourth, Ladizinsky^3^, based on chromosome pairing and hybridization with *A. sativa*, inferred that *A. insularis* was closer to hexaploids than any other known tetraploids. However, Jellen and Ladizinsky^58^ were unable to determine that *A. insularis* was the immediate ancestor of the hexaploid oats based on a comparative Giemsa-C banding investigation with *A. maroccana* and *A. murphyi*. Based on molecular diversity of the 5 S rRNA gene, Peng *et al*.^59^ revealed a finding favouring *A. insularis* as an AC genome donor to hexaploid oat. Also, Ladizinsky^60^ revealed some cytogenetic evidence that *A. insularis* did not contain the A-genome, and reasoned that it had either AC or CD genome. Fifth, *A. agadiriana* genome is unique among tetraploid species^7,61^ and has been favored to be distinguished from other so-called AB genome tetraploids^8,20,62^, as this species may contain an AD genome combination. Leggett^63^ reported 19 univalents per pollen mother cell in interspecific hybrids with AC genome *A. maroccana*. Several studies showed its genome similarities with AC tetraploids^19,64^ and some hexaploid oats^62^. Yan *et al*.^4^ recently reported that *A. agadiriana* was evolutionarily unique and was closer to Al genome *A. longiglumis* and AC genome tetraploids. Sixth, some karyological studies showed that Cv genome *A. ventricosa* and Al genome *A. longiglumis* had structurally altered derivative karyotypes^65^ and that *A. canariensis* and *A. longiglumis* had similar C-banding patterns with some differences in size and morphology of several chromosomes^66^. The karyotypic variations were presumably resulted from structural rearrangements during the divergence of C or A genomes from their common ancestors^67^. Reasoning based on the results of interspecific crosses, Loskutov and Rines (page 133)^10^ concluded that the tetraploids bearing the AC-genomes originated from diploid species bearing its components, presumably *A. canariensis* and *A. ventricosa*.

However, empirical evidence inconsistent with or against the proposed maternal pathway is not lacking. For example, morphological traits and habitats known for *A. venticosa* are not in favor for this plant as the progenitor of the known AC genome tetraploids^7^. Al genome *A. longiglumis* was placed in a cp-based clade of *Avena* species far from the clade with *A. insularis* and *A. sativa* (see Figure 6 of Liu *et al*.^19^). Also, our acquisition of organelle genomic signals from one sample per species may have had weakness in the inference of species or genome relatedness^19^. Using only the cp- or mt-specific SNP information might also carry some bias in the inferences of closely related species and estimations of species divergences. Moreover, the maternal pathway can provide only a complementary view of oat evolution, as it may not necessarily reflect the true nuclear genome origins towards hexaploid oat. Sampling bias might also occur, as our *Avena* samples (Table 1) may not represent well the biogeographical distribution of *Avena* species, particularly in the Morocco-Spain region. For example, the important *A. hirtula* was absent in this assay. Many *Avena* species may have gone extinct over 20 million years of evolution in the region^7^. Clearly more research is needed for continuous exploration on new and extinct oat species with an incorporation of biogeographical and historical variables.

### Dating species divergences

Our divergence dating calibrated based wheat-oat divergence of 25 Mya revealed the crown ages of several major divergences within *Avena* species. For example, C genome oat species diverged around 20 Mya from the remaining oat species. These dating results are consistent with those reported using nucleotide diversity of a few cp genes^19^. For example, both dating estimations showed a similar divergence of *A. ventricosa* from C genome species around 10–11 Mya and a separation of *A. canariensis* from the other A genome species around 13–15 Mya. Our dating results are also compatible with those done based on sequence variation of 5 S rRNA genes^59^ and of vitamin E biosynthesis genes^50^ and those cp-based divergence inferences in Triticeae^68,71^. For example, the most recent common ancestor of all Triticeae was estimated to be between 10 and 19 Mya^71^. However, our dating analysis also revealed the limit of using genome-wide SNP variability of organelle genomes to infer divergences among specific related species. For example, using cp SNP data, the youngest species divergence was estimated to be 3.90 Mya between *A. brevis* and *A. hispanica*. Similarly, *A. sativa* was estimated to separate from *A. sterilis* 4.9 Mya. In contrast, using sequence variation of specific cp genes, Liu *et al*.^19^ reported the crown ages of the *A. sativa* lineages ranging from 2.43 to 2.97 Mya. The revealed limitation of organelle-based dating is not surprising, given the known impacts of ancestral population size and incomplete lineage sorting on Bayesian estimation of species divergence times with substantial age overestimation^68–71^. For example, Middleton *et al*.^68^ argued that splits of chloroplast lineages might be older than the respective grass species, resulting in overestimated taxon ages for young clades.

### Practical implications

Oat wild relatives are known to harbor an important source of genetic variability for oat genetic improvement through interspecific crossing and introgression^10,25,72^, and documentation is not lacking on the successful interspecific transfer of alleles conferring resistance to disease^73,74^. Our oat phylogenetic analysis has provided information useful for identification and exploration of the closely related wild relatives for cultivated oats. Such information will allow for the better understanding of interspecific fertility barriers, and enhance the search for useful genes and the introgression of these genes from oat wild relatives to oat breeding programs.

Our phylogenetic analysis also revealed some uncertainties in the inference of evolutionary pathway towards hexaploid oats. For example, *A. insularis*, although the oldest AC genome tetetraploid, may not necessarily be the actual AC genome donor for cultivated hexaploid oat^58^. To address these uncertainties requires considerable efforts to collect oat wild relatives in their geographic distributions and to identify oat genomes from wild relatives, particularly for those species closely related to ACD genome species. Such field collection is not only to help facilitate the inference of oat genome relationships, but also to conserve important germplasm for future oat improvement^7,25^.

The successful application of multiplexed shotgun sequencing to acquire phylogenetic signals from organelle genomic variability is encouraging for exploring maternal lineages of crop wild relatives in several aspects^30^. First, the plastid extraction and multiplexed shotgun sequencing were not only effective to acquire cp, but also mt, genomic variability, allowing for the consistency assessment of phylogenetic signals from different organelles. Second, the separations of unique cp and mt sequence reads with the 3GenomeSNP pipeline were also effective, and the SNP calls based on allele frequencies estimated using genotype likelihoods by the ANGSD pipeline are expected to be less biased than those by other bioinformatics pipelines^30^. Third, the total laboratory and sequencing cost for sequencing 25 *Avena* samples was roughly $14,000, implying the feasibility of its wider application in acquisitions of genome-wide cp or mt SNPs from crop wild relatives for genetic and phylogenetic analyses.

## Electronic supplementary material

Supplementary Material
