# Supplementary material for: Oat evolution revealed in the maternal lineages of 25 Avena species
Source: Sci Rep. 2018 Mar 9;8:4252. doi: 10.1038/s41598-018-22478-4 (PMC5844911; doi:10.1038/s41598-018-22478-4)

Supplementary information for Scientific Reports

(DOI: 10.1038/s41598-018-22478-4)

Oat evolution revealed in the maternal lineages of 25 *Avena* species

Yong-Bi Fu

Plant Gene Resources of Canada, Saskatoon Research and Development Centre, Agriculture and Agri-Food Canada, 107 Science Place, Saskatoon, SK S7N 0X2, Canada. Phone: 306-385-9298, Fax: 306-385- 9489, email: yong-bi.fu@agr.gc.ca

Supplementary information for SNP calling

SNP calls were conducted using the ANGSD pipeline with the following parameters as example: angsd -bam bam.filelist -GL 2 -out gatk_outfile -doMaf 2 -doMajorMinor 1 -SNP_pval 1e-6 -doGeno 5 -doPost 1 -postCutoff 0.95.

Supplementary information for phylogenetic analyses

The specific parameters or options used to run different phylogenetic software are given below. Note that the options and parameters used below to generate MCC, MB, ML and MP trees were obtained from large training trials for each method.

1. Using BEAST software with the final BEAST settings as example:

The substitution model was K80 (HKY); clock model was relaxed clock exponential; tree prior was Yule model; the outgroup was wheat cp or mt for monophyletic analysis with prior of Inverse gamma; and the rest of options were kept with default values with a MCMC chain length of 50 millions.

2. Using MrBay with the following settings as example:

The substitution model included: lset applyto=(all) nst=2 rates=gamma ngammacat=4; prset applyto=(all) statefreqpr=fixed(equal). The priors consisted of prset brlenspr = unconstrained:exp(10.0) shapepr=exp(1.0) tratiopr=beta(1.0,1.0) statefreqpr = dirichlet(1.0,1.0,1.0,1.0). The MCMC parameters were: mcmcp ngen=2000000 samplefreq=100 printfreq=100 nruns=2 nchains=2 stoprule=yes stopval=0.01. The outputs included: sump burninfrac=0.25; sumt burninfrac=0.25.

3. Using RAxML with the K80 substitution model (-m GTRCAT -V --K80) and with the following executed parameters and options as example:

raxmlHPC-PTHREADS -T 16 -f a -m GTRCAT -V --K80 -p 112456 -x 85143 -s 26oatCP.phy -# 10000 -o wheat_CP -n 26oatCP-rmxl-og.tre.

4. Using PAUP* with the following parameters and options as example:

bootstrap nreps=5000 brlens=yes cutoffpct=0 treefile=26oatMT-boottrees.tre search=heuristic/ addseq=random nreps=10 swap=tbr hold=1.

Figure S1. Frequency distribution of the detected chloroplast (cp) and mitochondrial (mt) SNPs among 25 oat species in relation to the reference organelle genomes of wheat. **A**: all 6329 cp SNPs. **B**: 6343 mt SNPs. All these SNPs have no missing values across the 25 samples. The horizontal axis shows the position of the reference organelle genome of wheat, while the vertical axis shows the SNP count in each 100 or 300 bp window for the cp or mt genomes, respectively. The value in the horizontal axis of each peak or valley point (>0) was obtained by averaging all the SNP positions in the window.

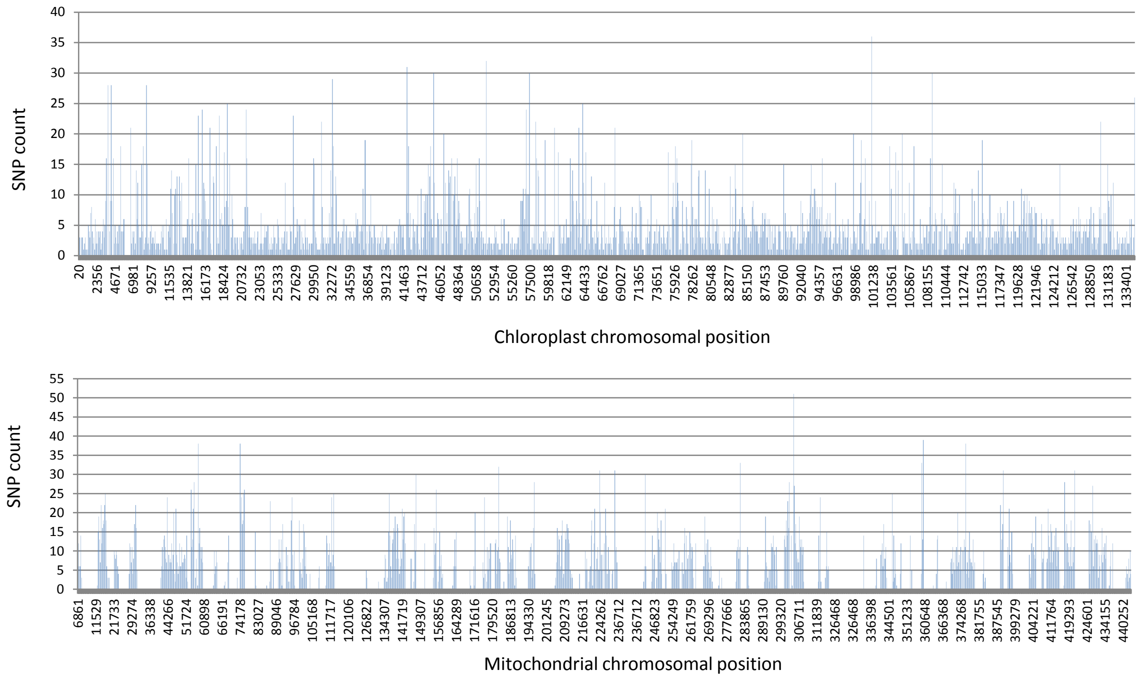

Figure S2. Minor allele frequency distributions for all 6329 chloroplast (cp) SNPs (**A**) and 6343 mitochondrial (mt) SNPs (**B**) detected in 25 oat species.

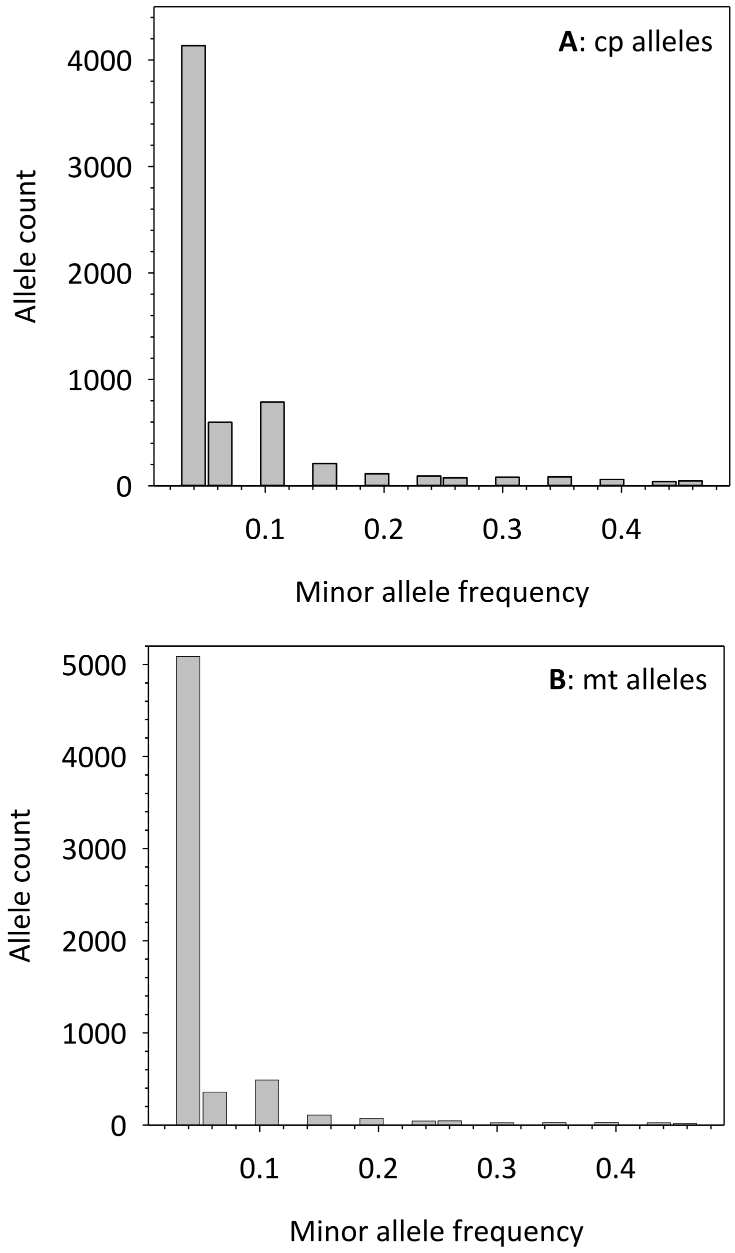

Figure S3. Phylogenetic trees of 25 oat species with branch support and wheat as an outgroup. They were inferred by MrBay (MB), RAxML (RM) and PAUP* (MP) methods based on 6329 chloroplast (cp), 6343 mitochondrial (mt) and 12,672 combined (cpmt) SNP data sets. The branch supports (in percentage) are posterior probability obtained from MrBay (**A-C**), maximum likelihood bootstrap values from RMxML (**D-F**), and bootstrap values from PAUP* (**G-I**).

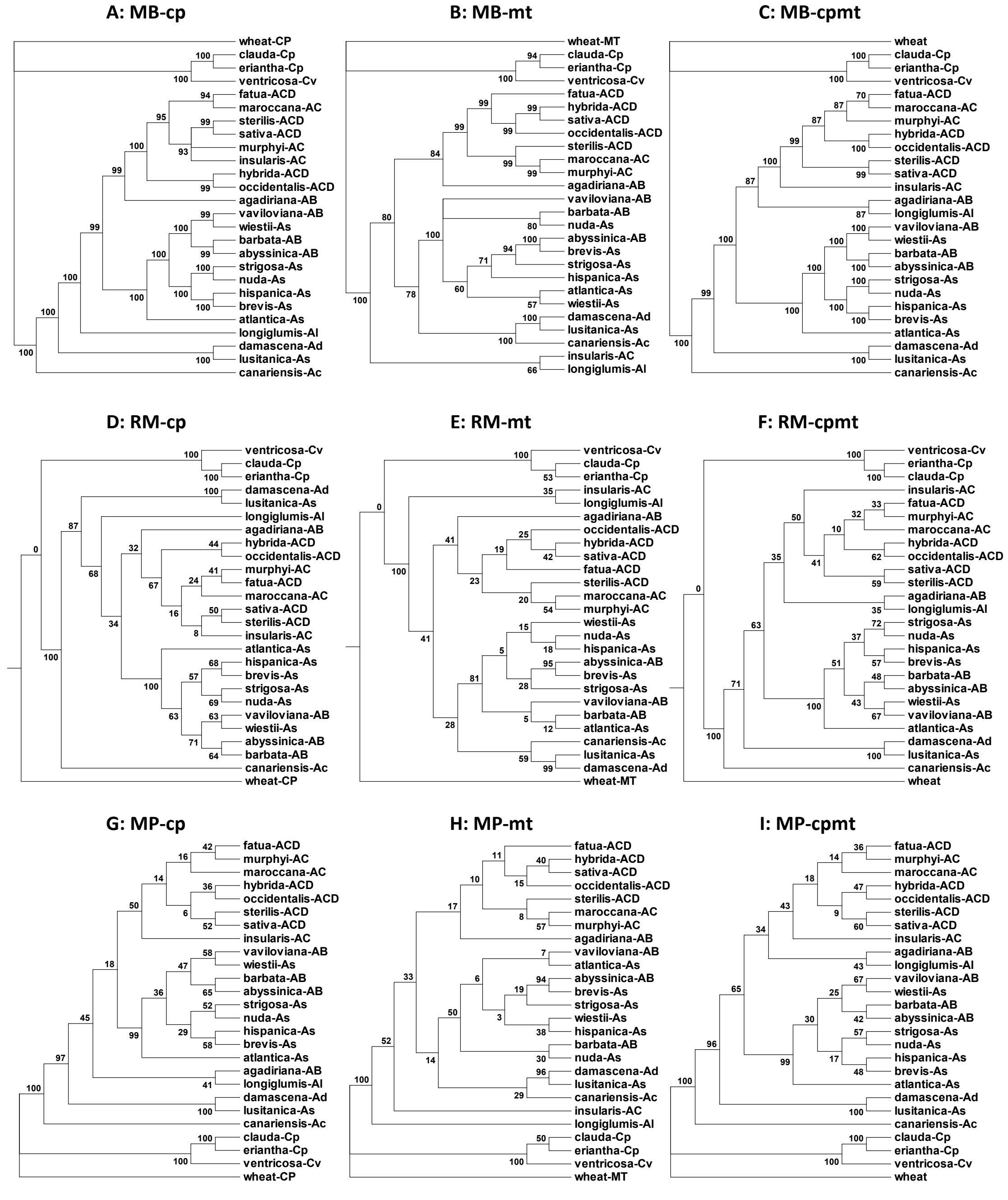

Figure S4 . Evolutionary maternal relationships of A and C genome species with ACD genome species. They were inferred using MrBay (MB), RAxML (ML) and PAUP* (MP) software based on 6329 chloroplast (cp), 6343 mitochondrial (mt) and 12,672 combined (cpmt) SNP data sets. A genome species were more related to ACD genome species *A. sativa* and *A. sterilis* than C genome species. *A. longiglumis* was the most closely related A genome species to *A. sativa*. Similarly, *A. ventricosa* appeared to be a little closer than the two other C genome species to *A. sativa*. Four species (*A. longiglumis*, *A. ventricosa*, *A. sativa* and *A. sterilis*) are highlighted in red branch.

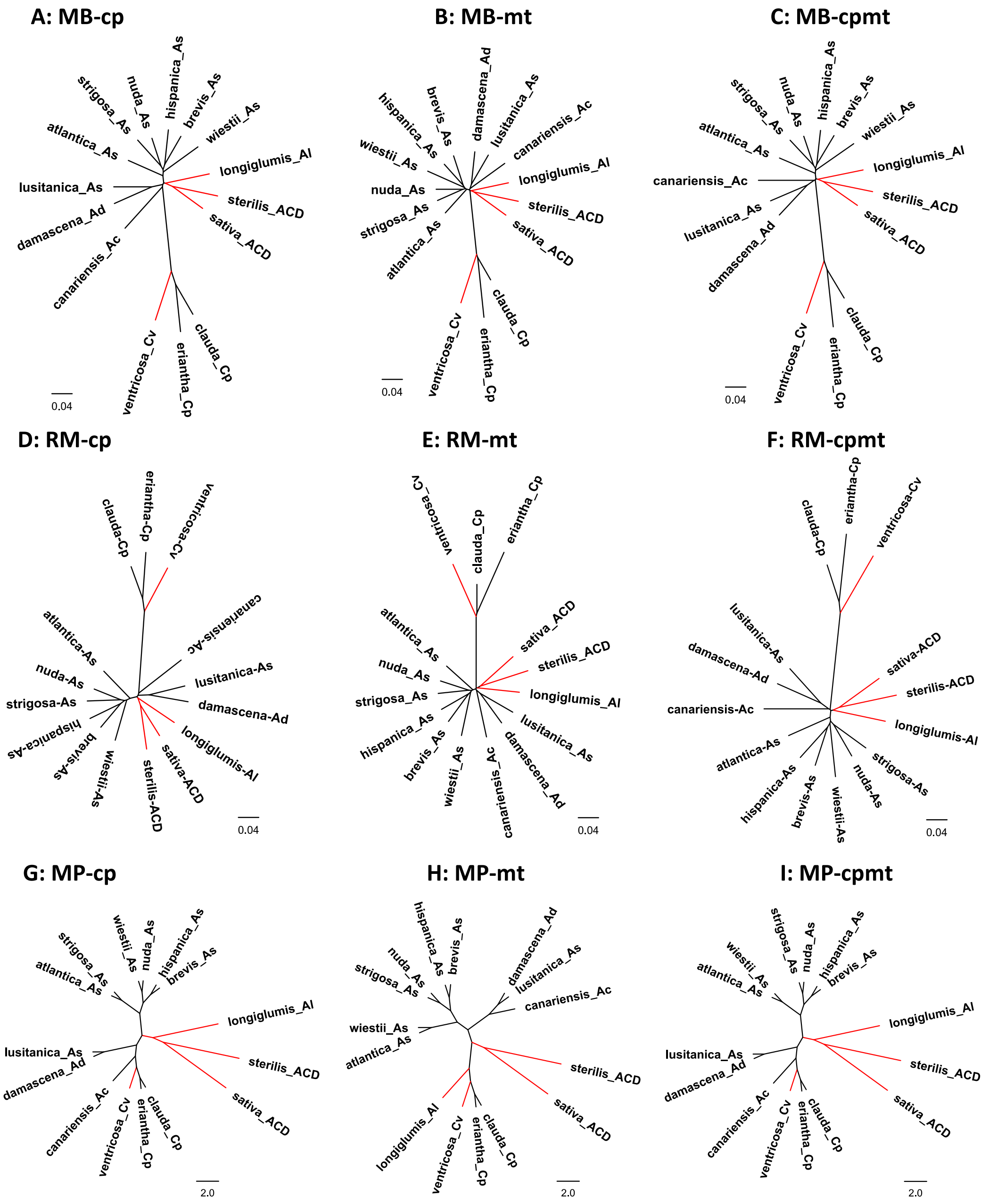

Figure S5. Evolutionary maternal relationships of A genome species with AC genome *A. insularis* and AB genome *A. agadiriana*. They were inferred using MrBay (MB), RAxML (ML) and PAUP* (MP) software based on 6329 chloroplast (cp), 6343 mitochondrial (mt) and 12,672 combined (cpmt) SNP data sets. Ac genome *A. canariensis* or Al genome *A. longiglumis* was closer to tetraploids of *A. insularis* and *A. agadiriana* than the other A genomes based on cp data. Based on cpmt data, *A. canariensis* was not closer to *A. insularis* and *A. agadiriana*. Four species (*A. canariensis*, *A. longiglumis*, *A. insularis* and *A. agadiriana*) are highlighted in red branch.

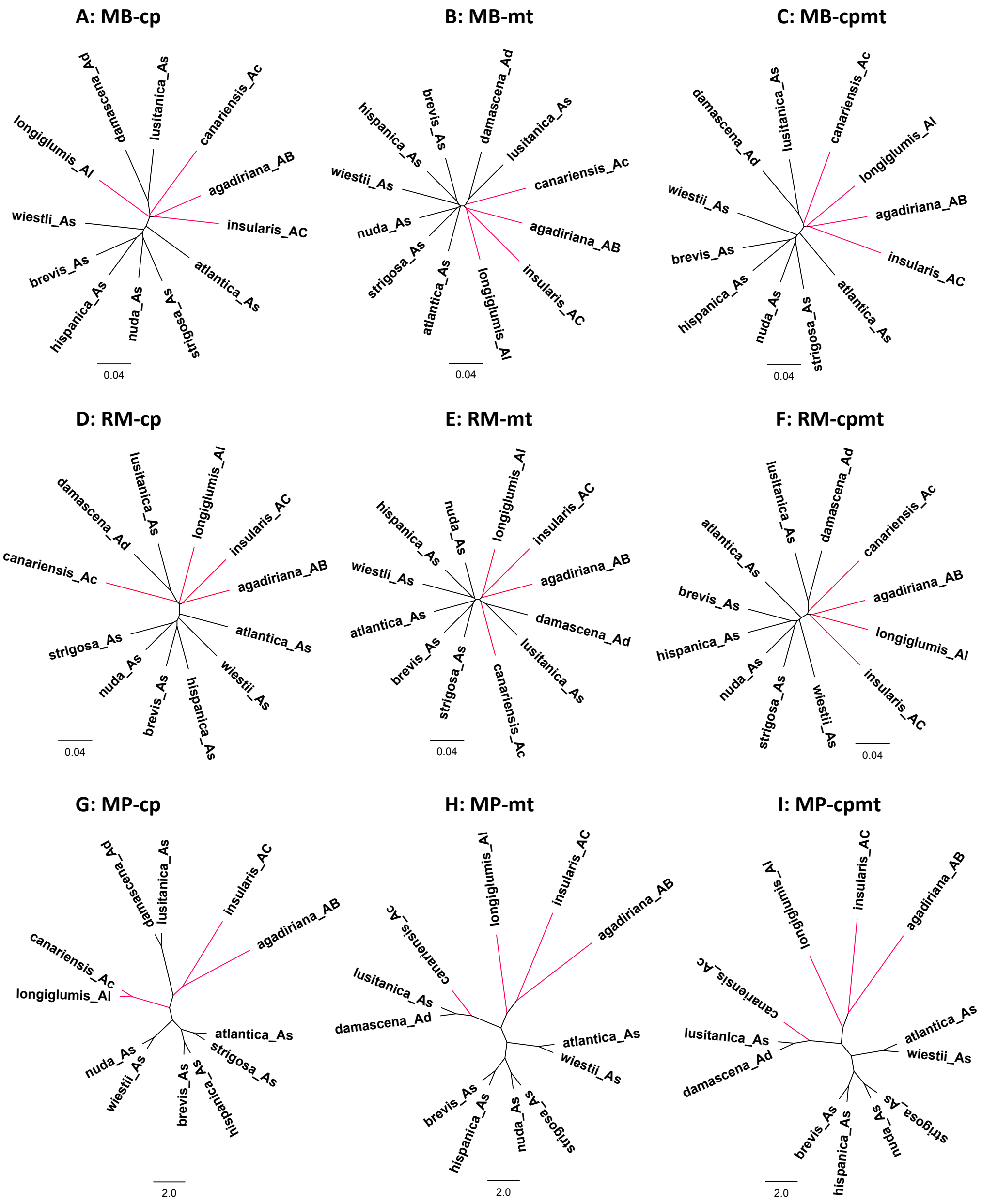

Figure S6. Evolutionary maternal relationships of AC and AB genome species with ACD genome species *A. sativa* and *A. sterilis*. They were inferred using MrBay (MB), RAxML (ML) and PAUP* (MP) software based on 6329 chloroplast (cp), 6343 mitochondrial (mt) and 12,672 combined (cpmt) SNP data sets. AC genome species were more closely related to ACD genome species, as highlighted in red branch, than AB genome species. Based on cpmt data, Al genome *A. longiglumis* and AB genome *A. agadiriana* were closely related and highlighted in red label.

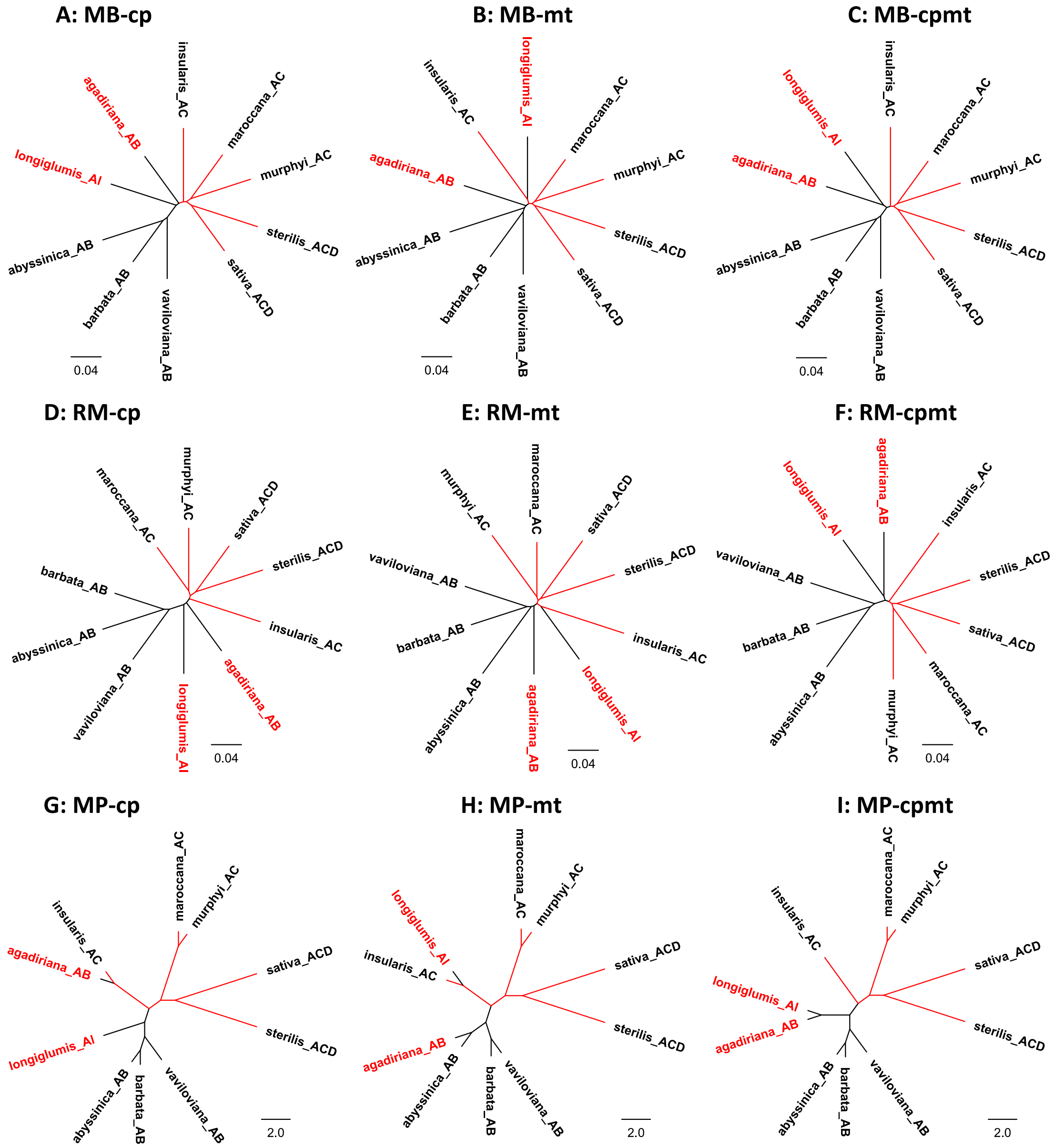

Supplement: Supplementary file 1 — Supplementary Material [file 41598_2018_22478_MOESM1_ESM.pdf]
